# State of the Art and New Perspectives in Lung Cancer Therapeutics

**DOI:** 10.3390/cancers16213631

**Published:** 2024-10-28

**Authors:** Kostas A. Papavassiliou, Amalia A. Sofianidi, Vassiliki A. Gogou, Athanasios G. Papavassiliou

**Affiliations:** 1 First University Department of Respiratory Medicine, ‘Sotiria’ Chest Hospital, Medical School, National and Kapodistrian University of Athens, 11527 Athens, Greece; konpapav@med.uoa.gr (K.A.P.); gogvanessa@gmail.com (V.A.G.); 2 Department of Biological Chemistry, Medical School, National and Kapodistrian University of Athens, 11527 Athens, Greece; amsof.00@gmail.com

Cisplatin became a first-line chemotherapy regimen for lung cancer in the mid-1980s, marking a pivotal advance in lung cancer treatment [1,2]. Since then, the therapeutic landscape has evolved significantly, with major breakthroughs in surgical techniques, chemotherapy regimens, precision medicine, immunotherapy, and targeted therapies. However, despite these innovations, the overall 5-year survival rate across all stages of lung cancer remains low, at just 19% [3]. Alarmingly, 57% of patients are diagnosed at an advanced stage, where the disease has already metastasized throughout the body [4]. These realities underscore an urgent need for cutting-edge therapies to combat this formidable disease.

Targeted therapies aim to counteract the molecular alterations driving lung cancer. While third-generation tyrosine kinase inhibitors (TKIs) have been effective, resistance inevitably develops over time [5]. BBT-176, a novel fourth-generation TKI currently undergoing clinical trials, has shown promising preliminary efficacy against the epidermal growth factor receptor (EGFR) C797S mutation—a key factor in the development of such resistance [6]. Recent progress has led to the development of small-molecule inhibitors like sotorasib that target Kirsten rat sarcoma (KRAS) mutations, previously considered “undruggable” [7]. Ongoing research aims to refine these drugs and expand direct or indirect treatment options for patients with other KRAS alterations [8]. Regarding anaplastic lymphoma kinase (ALK) inhibitors, SAF-189s is an innovative ALK inhibitor that has demonstrated potential in preclinical studies to overcome most known resistance mutations linked to ALK [9]. The latest additions to the armamentarium against lung cancer include a rapidly emerging class of therapeutics called antibody–drug conjugates, such as trastuzumab deruxtecan, which targets the human epidermal growth factor receptor 2 (HER2) [10]. The promise of targeted therapy lies in combining these molecules with each other and integrating them with other therapeutic approaches.

In the field of immunotherapy, the future of lung cancer treatment is being shaped by pioneering approaches such as lung cancer vaccines, adoptive cell transfer (ACT), and oncolytic viruses. Preventative vaccines for high-risk groups are anticipated in the coming years, aiming to reduce the incidence of the disease. Notably, this past August, the first lung cancer patient received an mRNA vaccine, heralding the beginning of a new era in therapeutic vaccines. ACT includes chimeric antigen receptor T (CAR-T) cells and tumor-infiltrating lymphocyte (TIL) therapy, with both currently being evaluated in clinical trials [11]. Dendritic cell (DC) vaccination has the potential to transform the tumor microenvironment (TME) in lung cancer patients, promoting sustained anti-tumor immune activity [12]. This approach may also offer the advantage of fewer adverse events, such as cytokine release syndrome (CRS), which is often associated with CAR-T cell therapy [13]. Oncolytic viruses operate through a dual mechanism: they selectively replicate within and destroy tumor cells that lack effective antiviral defenses, thereby stimulating a stronger antitumor adaptive immune response. Simultaneously, when oncolytic viruses infect healthy cells, they trigger antiviral reactions that help recruit immune cells to the TME, further enhancing the immune attack against tumor cells [14].

Recent advancements in radiation therapy have significantly improved its precision, efficacy, and safety, paving the way toward innovative future applications. Intensity-modulated radiotherapy (IMRT) and volumetric-modulated arc therapy (VMAT) are sophisticated radiation treatment techniques that enhance dosimetric results in lung cancer care by precisely focusing on tumors while protecting surrounding healthy tissue [15]. Stereotactic body radiotherapy (SBRT) administers high doses of radiation with remarkable accuracy to small, well-defined tumors [16]. Additionally, the highly promising boron neutron capture therapy (BNCT) leverages the selective accumulation of boron-10 compounds within cancer cells, allowing for the targeted destruction of these malignant cells [17]. Building on this, artificial intelligence (AI) offers a powerful tool for fostering cancer care. AI can improve the accuracy of tumor identification, optimize radiation dosing, and even predict patient responses to treatment. By exploiting machine learning algorithms to analyze extensive patient data, personalized treatment plans can be developed, leading to better outcomes and fewer side effects. Notably, radiomics—through the analysis of imaging features—offers deeper insights into tumor biology and behavior [18], heralding a new era of tailored and effective radiation therapies.

Various types of nanoparticles have recently emerged as powerful tools for enhancing cancer drug delivery in both preclinical studies and clinical trials, offering the promise of controlled and sustained release [19]. Lipid-based nanoparticles, such as liposomes, solid lipid nanoparticles, and nanostructured lipid carriers, are known for their biocompatibility and capacity to encapsulate both hydrophilic and hydrophobic drugs. Polymeric nanoparticles, including micelles, dendrimers, and nanogels, provide versatility in drug loading and sustained release. Inorganic nanoparticles, like gold, silver, and iron oxide nanoparticles, as well as quantum dots, are valued for their stability and potential use in targeted therapies. Additionally, hybrid nanoparticles, which combine organic and inorganic components, offer enhanced functionality and precision, making them promising candidates for more effective and targeted lung cancer treatments [8,19].

In conclusion, the therapeutic landscape of lung cancer treatment is moving from a one-size-fits-all approach to a more personalized one. The development of predictive biomarkers, combined with the valuable support of AI, has the potential to provide lung cancer patients with more tailored and effective treatments, ultimately improving their quality of life. Nevertheless, it is crucial to address the risk of widening disparities in cancer care, as personalized approaches could come with higher costs, potentially limiting access for some patients.
